# Investigation of the status of depression and anxiety among leaders and staff in pharmacy intravenous admixture services in China

**DOI:** 10.1097/MD.0000000000034661

**Published:** 2023-08-25

**Authors:** Chunsong Yang, Dan Li, Yaya Yang, Yong Hu, Lingli Zhang

**Affiliations:** a Department of Pharmacy, Evidence-based Pharmacy Center, West China Second Hospital, Sichuan University, Chengdu, China; b Key Laboratory of Birth Defects and Related Diseases of Women and Children (Sichuan University), Ministry of Education, Chengdu, China; c Department of Pediatric Clinic, West China Second Hospital, Sichuan University, Chengdu, China; d Department of Pediatrics, West China Second University Hospital, Sichuan University, Chengdu, China.

**Keywords:** anxiety, depression, PIVAS, questionnaire survey

## Abstract

We aimed to investigate the status of depression and anxiety among leaders and staff in pharmacy intravenous admixture services (PIVAS) and analyze influencing factors to provide a reference for improving their mental health status. This study involved a multi-center cross-sectional survey. PIVAS leaders and staff from across China were invited to participate. The Generalized Anxiety Disorder-7 and 9-item Patient Health Questionnaire were used to measure the status of anxiety and depression. A multiple linear regression model was used to analyze influencing factors. In total, 137 PIVAS leaders and 501 staff were included in this study. The results showed that 43.8% (60/137) of the leaders had anxiety and 38.7% (53/137) had depression. Among staff, 47.3% (237/501) had anxiety and 46.5% (233/501) had depression. Multiple linear regression showed that for PIVAS leaders, the degree of recognition by doctors was associated with anxiety scores, whereas PIVAS charge standard, PIVAS profit and loss situation, and the degree of recognition by doctors were associated with depression scores. For PIVAS staff, satisfaction with working in PIVAS was associated with anxiety scores, and job title, working hours per day, and satisfaction with working in PIVAS were associated with depression scores. Depression and anxiety are common among PIVAS leaders and staff working in hospitals in China. Hospitals should implement measures to improve the mental health of PIVAS leaders and staff.

## 1. Introduction

Pharmacy intravenous admixture services (PIVAS) refer to hospital based comprehensive and technical pharmaceutical departments that integrate clinical pharmacy and scientific research according to international standards and good manufacturing practice standards. PIVAS pharmacists review medical orders and perform centralized admixture of intravenous drugs (e.g., general drugs, tumor chemotherapy drugs, antibiotics, and total parenteral nutrition) in accordance with sterile operation standards and in clean environmental conditions to provide intravenous infusions for patients.^[1–3]^

The rapid growth of intravenous infusion therapy in recent years means the workload associated with centralized admixture of intravenous drugs in PIVAS has increased. Because this work is important and specialized, the requirements for PIVAS staff and leaders are relatively high. For example, they must have professional knowledge and skills, as well as a high degree of responsibility, a serious and careful work attitude, and good communication skills.^[4–6]^ The busy daily work and pressure burden mean that staff may be prone to anxiety and depression, which affects their quality of life and work, and reduces their daily work efficiency and accuracy of medication dispensing, which poses a serious threat to medical safety in hospitals.

It is necessary to understand the mental health status of PIVAS staff to guarantee medical safety and quality. However, few studies have focused on this topic. In 2019, Chao et al^[7]^ investigated 239 nurses working in PIVAS in 3 cities in Shaanxi Province in China and analyzed their current status and factors influencing anxiety. They found a high rate of anxiety among nurses working in PIVAS. However, that study only included participants from 1 province, focused on anxiety status, and did not consider depression status, meaning the research results are not representative. In 2015, Jin et al^[8]^ analyzed the mental health status of 40 nurses working in PIVAS, but that sample size was small, and they did not analyze factors influencing mental health status.

Therefore, we conducted a multi-center cross-sectional survey in China to investigate the status of depression and anxiety among PIVAS leaders and staff, and analyze the factors influencing depression and anxiety to provide a reference for improving their mental health status.

## 2. Methods

### 2.1. Study design

This was a multi-center cross-sectional survey. We used a self-designed questionnaire to investigate the status of depression and anxiety among leaders and staff working in PIVAS in China. We distributed the questionnaire via the WeChat group for the Intravenous Dispensing Management and Application Branch of the China Medical Education Association, there are 324 members in the group. We also ask the PIVAS leaders to invite their colleagues to attend this survey via a face-to-face invitation.

### 2.2. Participants

All leaders and staff working in PIVAS across China were invited to participate in this survey. The inclusion criteria were as follows: leaders and staff were working in PIVAS across China; the job titles of participants were junior or above; participants were willing to attend this survey. The exclusion criteria were as follows: participants refused to participate in the survey; participant was an intern or a trainee; and participants have no job titles.

### 2.3. Information collected

This study used a self-designed questionnaire to collect data to investigate the status of anxiety and depression of PIVAS leaders and staff. The questionnaire collected basic information for leaders (gender, region, hospital level, job title) and staff (gender, region, hospital level, job title, education background, working years, working hours per day, whether PIVAS training was sufficient, importance PIVAS attached to personnel training, satisfaction with PIVAS training, satisfaction with working in PIVAS, whether they had mastered scientific research methodology, need for scientific research, interest in scientific research, and the ability to do scientific research). The questionnaire also collected basic information about PIVAS, including daily average infusion volume, PIVAS charge standard, PIVAS profit and loss situation, degree of recognition by doctors, degree of recognition by nurses, need for scientific research among staff, interest in scientific research among staff, whether staff had mastered scientific research methodology, and staff scientific research ability.

### 2.4. Outcome measurement

The Generalized Anxiety Disorder-7 (GAD-7) was used measure the status of anxiety.^[9]^ Each item describes 1 typical generalized anxiety disorder symptom and is evaluated by the frequency with which that symptom was reported over the last 2 weeks. Each item is scored from 0 (not at all) to 3 (nearly every day). Item scores are summed to give a total GAD-7 score ranging from 0 to 21. A score of 0–4 is defined as no anxiety, 5–9 as mild anxiety, 10–13 as moderate anxiety, 14–18 as moderate to severe anxiety, and 19–21 as severe anxiety.

The 9-item Patient Health Questionnaire (PHQ-9) was used to measure participants’ depression status.^[10]^ The PHQ-9 focuses on the frequency of occurrence over the past 2 weeks of 9 depressive symptoms derived from the Diagnostic and Statistical manual of Mental Disorders, Fourth Edition diagnostic criteria. Each item is scored from 0 (not at all) to 3 (nearly every day), with the total score ranging from 0 to 27. A PHQ-9 score of 0–4 is defined as no depression, 5–9 points as mild depression, 10–14 points as moderate depression, and 20–27 as severe depression. We used the Chinese version of GAD-7 and PHQ-9 to conduct the outcome measurement.

### 2.5. Quality control

Before the formal investigation, we improved the contents of the questionnaire by conducting pre-investigation. During the investigation, we strictly followed the inclusion and exclusion criteria, selected the participants, and checked the rationality of the questionnaire in time. After the investigation, we checked the completeness and authenticity of the results of investigation.

### 2.6. Statistical methods

Normally distributed data were expressed as mean ± standard deviation, and t-tests or analysis of variance was used for the analysis. A rank sum test was used when the data were not normally distributed. Categorical variables were analyzed with chi-square tests. Following a univariate analysis, factors with a *P* value ≤ .2 were included in the multiple linear regression model. *P* values ≤ .05 were considered statistically significant. We used SPSS version 21.0 (IBM Corp., Armonk, NY) for the data analyses.

## 3. Results

### 3.1. Participants’ characteristics

#### 3.1.1. PIVAS leaders.

In total, 137 PIVAS leaders were included in this study, the response rate was 42.3%. 39.4% (54/137) were male and 60.6% (83/137) were female. Over half (56.2%, 77/137) were located in eastern China, 27.0% (37/137) were in western China, and 6.8% (23/137) were in central China. The majority of leaders worked in Level III Grade A hospitals (74.5%, 102/137) and (13.9%, 19/137) worked in Level III Grade B hospitals. Participating leaders’ job titles included: senior titles (12.4%, 17/137), deputy titles (45.3%, 60/137), intermediate titles (37.2%, 51/137), and junior titles (5.1%, 7/137).

#### 3.1.2. PIVAS staff.

Overall, 501 PIVAS staff were included in this study; 24.2% (121/501) were male and 75.8% (380/501) were female. Over half of these participants (56.1%, 281/501) were located in eastern China, 27.5% (138/501) were in western China, and 16.4% (82/501) were in central China. Most PIVAS staff worked in Level III hospitals (91.0%, 456/501). The largest group of participants had junior job titles (49.5%, 248/501) followed by intermediate job titles (22.2%, 111/501). The majority of staff held a bachelor’s degree (72.5%, 363/501). Their work experience in PIVAS ranged from 0.08 to 35 years (mean 4.26 ± 3.56 years).

### 3.2. Prevalence of anxiety and depression

#### 3.2.1. PIVAS leaders.

The anxiety scores for PIVAS leaders ranged from 1 to 10, with a mean score of 5.26 ± 4.41 (median: 4). Overall, 56.2% (77/137) of leaders had no anxiety, 29.2% (40/137) had mild anxiety, and 14.6% (20/137) had moderate anxiety. The depression scores ranged from 0 to 18, with a mean score of 3.87 ± 3.78 (median: 2); 61.3% (84/137) of leaders had no depression, 29.9% (41/137) had mild depression, and 8.8% (12/137) had moderate depression.

#### 3.2.2. PIVAS staff.

Among PIVAS staff, the anxiety scores ranged from 1 to 24, with a mean score of 5.47 ± 4.89 (median: 4). Overall, 52.7% (264/501) had no anxiety, 32.1% (161/501) had mild anxiety and 15.2% (76/501) had moderate anxiety. Depression scores ranged from 0 to 24, with a mean score of 4.92 ± 4.44 (median: 4); 53.5% (268/501) had no depression, 32.9% (165/501) had mild depression, and 13.6% (68/501) had moderate depression.

### 3.3. Factors affecting anxiety and depression

#### 3.3.1. PIVAS leaders.

In the univariate analysis, statistical test of 4 variables showed that *P* value was less than 0.2: PIVAS profit and loss situation (*P* = .111), degree of recognition by doctors (*P* = .14), interest in scientific research among staff (*P* = .049), and staff scientific research ability (*P* = .048). We included factors with a *P* value < .20 in the univariate analyses in our multiple linear regression model. The results showed that only the degree of recognition by doctors (β = −0.170; standard error [SE] = 0.656; *P* = .049) was associated with anxiety scores (Table 1).

**Table 1:** Univariate linear regression analysis of factors influencing anxiety and depression of PIVAS leaders.

Item	Option	n	Anxiety score	*F*	*P*	Depression score	*F*	*P*
Gender	Male	54	5.04 ± 4.296	0.218	.642	3.70 ± 4.022	0.169	.682
	Female	83	5.40 ± 4.499			3.98 ± 3.626		
Region	Eastern	77	5.39 ± 4.609	0.110	.896	3.94 ± 3.760	0.510	.602
	Western	37	4.97 ± 4.003			4.16 ± 3.862		
	Central	23	5.26 ± 4.505			3.17 ± 3.762		
Hospital level*	Level III Grade A hospital	102	5.07 ± 4.528	0.455	.636	3.71 ± 3.674	0.417	.660
	Level III Grade B hospital	19	6.11 ± 3.928			4.53 ± 4.312		
	Level II Grade A hospital	16	5.44 ± 4.289			4.13 ± 3.897		
Job title	Senior title	17	6.71 ± 5.169	0.786	.503	5.82 ± 3.592	1.869	.138
	Deputy title	62	5.18 ± 4.682			3.42 ± 3.462		
	Intermediate title	51	5.00 ± 3.779			3.75 ± 3.979		
	Junior title	7	4.29 ± 4.424			4.00 ± 4.655		
PIVAS daily average infusion volume	0-1000	41	5.07 ± 4.338	0.389	.817	4.24 ± 4.055	0.514	.725
	1001-2000	36	5.58 ± 4.924			4.28 ± 3.716		
	2001-3000	19	5.95 ± 4.288			3.68 ± 3.845		
	3001-4000	21	5.29 ± 3.783			3.24 ± 3.448		
	≥4001	20	4.35 ± 4.545			3.20 ± 3.722		
PIVAS charge standard	Yes	72	4.85 ± 4.487	1.305	.255	3.35 ± 3.569	2.937	.089
	No	65	5.71 ± 4.307			4.45 ± 3.937		
PIVAS profit and loss situation	Profit	10	4.90 ± 3.900	4.388	.111	4.80 ± 5.789	3.798	.15
	Loss	96	5.81 ± 4.749			4.14 ± 3.652		
	Balance	31	3.65 ± 2.893			2.74 ± 3.235		
Degree of recognition by doctors	Disagree	6	6.33 ± 4.320	3.937	.14	6.67 ± 3.559	5.804	.004
	General	43	6.53 ± 5.184			5.02 ± 3.642		
	Agree	88	4.56 ± 3.865			3.11 ± 3.656		
Degree of recognition by nurses	Disagree	5	7.20 ± 4.207	0.543	.582	6.00 ± 4.301	0.894	.412
	General	13	4.85 ± 4.506			4.15 ± 3.997		
	Agree	119	5.22 ± 4.421			3.75 ± 3.735		
Need for scientific research among staff	Low	13	5.62 ± 5.455	0.882	.954	3.38 ± 3.709	0.142	.868
	General	53	5.43 ± 4.975			3.83 ± 3.583		
	High	71	5.06 ± 3.764			3.99 ± 3.996		
Interest in scientific research among staff	Low	34	6.24 ± 5.052	3.080	.049	3.65 ± 3.302	4.280	.118
	General	66	4.30 ± 4.132			3.23 ± 3.396		
	High	37	6.05 ± 3.993			5.22 ± 4.510		
Whether staff had mastered scientific research methodology	Few staffs	98	5.63 ± 4.510	3.013	.222	4.15 ± 3.602	1.045	.355
	Half staffs	4	5.50 ± 7.724			2.50 ± 3.317		
	Most staffs	35	4.17 ± 3.577			3.23 ± 4.25		
Staff scientific research ability	Low	71	6.10 ± 4.508	3.112	.048	4.39 ± 3.635	5.343	.069
	General	57	4.18 ± 4.192			2.95 ± 3.335		
	High	9	5.44 ± 3.844			5.56 ± 6.085		

PIVAS = pharmacy intravenous admixture services.

* In China, hospital level is an index to evaluate hospital qualifications according to the comprehensive level of hospital functions, tasks, facilities, technical construction, medical service quality and scientific management, and it is a national unified standard. According to the “Hospital Classification Management Standard,” the hospital is determined to be three levels, each level is further divided into A, B and C, a higher level of hospital means stronger strength.

Five variables (*P* < .2) may have associations with depression scores in the univariate analysis: job title (*P* = .138), PIVAS charge standard (*P* = .089), degree of recognition by doctors (*P* = .004), staff interest in scientific research (*P* = .118), and staff scientific research ability (*P* = .069). The multiple linear regression model revealed that PIVAS charge standard (β = −0.190; SE = 0.577; *P* = .020), PIVAS profit and loss situation (β = −0.243; SE = 0.540; *P* = .004), and the degree of recognition by doctors (β = 0.230; SE = 0.456; *P* = .010) were associated with depression scores (Table 2).

**Table 2:** Multiple linear regression analysis of factors influencing anxiety and depression of PIVAS leaders.

Variable	Non-standardized coefficient	Standardized error	Standardized coefficient	*t*	*P*
Anxiety					
Constant	12.398	2.480	–	5.000	.000
PIVAS profit and loss situation	−1.321	0.705	−0.158	−1.874	.063
Degree of recognition by doctors	−1.303	0.656	−0.170	−1.986	.049
Interest in scientific research among staff	0.351	0.553	0.058	0.635	.526
Staff scientific research ability	−1.048	0.653	−0.147	−1.607	.111
Depression					
Constant	−0.570	0.399	−0.115	−1.430	.155
Job title	1.055	0.610	0.140	1.730	.086
PIVAS charge standard	−1.361	0.577	−0.190	−2.358	.020
PIVAS profit and loss situation	−1.595	0.540	−0.243	−2.953	.004
Degree of recognition by doctors	1.201	0.456	0.230	2.632	.010
Interest in scientific research among staff	−0.588	0.536	−0.096	−1.098	.274
Staff scientific research ability	−0.570	0.399	−0.115	−1.430	.155

PIVAS = pharmacy intravenous admixture services.

#### 3.3.2. PIVAS staff.

In the univariate analysis, 6 variables had statistically significant associations with anxiety scores: working hours per day (*P* = .015), whether PIVAS training was sufficient (*P* = .047), importance PIVAS attached to personnel training (*P* = .047), satisfaction with PIVAS training (*P* = .000), satisfaction with working in PIVAS (*P* = .000), and interest in scientific research (*P* = .097). The results of the multiple linear regression model showed that only satisfaction with working in PIVAS (β = −0.400; SE = 0.406; *P* = .000) was associated with anxiety scores (Table 3).

**Table 3:** Univariate linear regression analysis of factors influencing anxiety and depression of PIVAS staff.

Item	Option	n	Anxiety score	*F*	*P*	Depression score	*F*	*P*
Gender	Male	121	5.77 ± 5.655	−0.021	.983	5.05 ± 5.125	−0.414	.679
	Female	380	5.37 ± 4.623			4.88 ± 4.408		
Region	Eastern China	281	5.45 ± 5.098	1.224	.295	5.05 ± 4.569	1.018	.362
	Western China	138	5.09 ± 4.173			4.47 ± 4.094		
	Central China	82	6.16 ± 5.248			5.22 ± 4.56		
Hospital level	Level III hospital	456	5.48 ± 4.899	0.010	.921	4.96 ± 4.451	0.419	.518
	Level II hospital	45	5.4 ± 4.84			4.51 ± 4.373		
Job title	Senior title	8	3.88 ± 3.137	1.210	.303	4.63 ± 3.543	2.396	.037
	Deputy title	25	4.88 ± 4.64			3.08 ± 2.798		
	Intermediate title	111	5.1 ± 4.675			4.26 ± 4.528		
	Junior title	248	5.61 ± 4.869			5.36 ± 4.482		
	Pre-Junior title	95	5.43 ± 5.224			4.77 ± 4.442		
	None	14	8.07 ± 5.54			6.79 ± 4.677		
Education background	Doctorate degree	4	4.75 ± 3.5	0.272	.846	4.5 ± 3.317	0.211	.889
	Master’s degree	31	5.77 ± 4.965			4.77 ± 4.08		
	Bachelor degree	363	5.36 ± 4.771			4.85 ± 4.324		
	College and below	103	5.79 ± 5.348			5.22 ± 5.002		
Working years			−1.595	0.111	–	−1.149	0.251	–
Working hours per day	≤6 h	7	2.71 ± 1.799	10.488	.015	2.29 ± 2.289	12.630	.006
	6–8 h	443	5.29 ± 4.746			4.69 ± 4.238		
	9–10 h	42	7.88 ± 6.145			7.38 ± 5.691		
	≥10 h	9	5.33 ± 3.808			6.67 ± 5.148		
Whether PIVAS training was sufficient	Insufficient	61	5.98 ± 4.455	3.081	.047	5.43 ± 4.241	3.669	.026
	General	157	6.12 ± 5.350			5.57 ± 4.831		
	Sufficient	283	5.00 ± 4.670			4.45 ± 4.212		
Importance PIVAS attached to personnel training	Not important	53	7.04 ± 5.046	3.079	.047	6.45 ± 4.330	4.038	.018
	General	147	5.26 ± 4.699			5.03 ± 4.452		
	Important	301	5.30 ± 4.919			4.60 ± 4.411		
Satisfaction with PIVAS training	Unsatisfactory	31	7.77 ± 6.412	28.362	.000	6.94 ± 5.910	24.089	.000
	General	241	6.29 ± 5.036			5.68 ± 4.613		
	Satisfied	229	4.29 ± 4.182			3.85 ± 3.738		
Satisfaction with working in PIVAS	Unsatisfactory	44	11.02 ± 6.308	70.010	.000	9.89 ± 5.040	80.356	.000
	General	225	6.03 ± 4.786			5.56 ± 4.353		
	Satisfied	232	3.88 ± 3.661			3.35 ± 3.468		
Whether they had mastered scientific research methodology	Yes	30	5.20 ± 4.972	1.432	.240	4.80 ± 5.176	1.553	.213
	No	309	5.76 ± 4.980			5.19 ± 4.214		
	Unclear	162	4.97 ± 4.682			4.43 ± 4.524		
Need for scientific research	Low	140	5.93 ± 5.307	1.175	.310	5.41 ± 4.512	2.178	.114
	General	237	5.44 ± 4.806			4.97 ± 4.649		
	High	124	5.01 ± 4.536			4.27 ± 3.877		
Interest in scientific research	Low	88	6.44 ± 5.377	2.340	.097	6.15 ± 4.820	4.952	.007
	General	251	5.39 ± 4.881			4.88 ± 4.425		
	High	162	5.07 ± 4.575			4.31 ± 4.138		
The ability to do scientific research	Low	276	5.84 ± 4.962	1.863	.156	5.29 ± 4.418	2.952	.053
	General	198	4.96 ± 4.781			4.33 ± 4.385		
	High	27	5.44 ± 4.766			5.48 ± 4.775		

PIVAS = pharmacy intravenous admixture services.

The univariate analysis showed that factors associated with depression scores were job title (*P* = .037), working years (*P* = .111), working hours per day (*P* = .006), whether PIVAS training was sufficient (*P* = .026), importance PIVAS attached to personnel training (*P* = .018), satisfaction with PIVAS training (*P* = .000), satisfaction with working in PIVAS (*P* = .000), need for scientific research (*P* = .114), interest in scientific research (*P* = .007), and staff scientific research ability (*P* = .053). However, in the multiple linear regression model, 3 factors were statistically significant: job title (β = 0.113; SE = 0.215; *P* = .012), working hours per day (β = 0.090; SE = 0.462; *P* = .030), and satisfaction with working in PIVAS (β = −0.401; SE = 0.364; *P* = .000) (Table 4).

**Table 4:** Multiple linear regression analysis of factors influencing anxiety and depression of PIVAS staff.

Variable	Non-standardized coefficient	Standardized error	Standardized coefficient	*t*	*P*
Anxiety					
Constant	10.927	1.664	–	6.567	.000
Working hours per day	0.481	0.517	0.039	0.930	.353
Whether PIVAS training was sufficient	0.060	0.359	0.009	0.167	.867
Importance PIVAS attached to personnel training	0.605	0.363	0.084	1.665	.096
Satisfaction with PIVAS training	−0.215	0.461	−0.027	−0.466	.641
Satisfaction with working in PIVAS	−3.050	0.406	−0.400	−7.511	.000
Interest in scientific research	−0.171	0.295	−0.024	−0.579	.563
Depression					
Constant	8.183	1.773		4.615	.000
Job title	0.545	0.215	0.113	2.530	.012
Working years	−0.053	0.055	−0.043	−0.977	.329
Working hours per day	1.009	0.462	0.090	2.182	.030
Whether PIVAS training was sufficient	−0.113	0.326	−0.018	−0.347	.729
Importance PIVAS attached to personnel training	0.199	0.327	0.030	0.609	.543
Satisfaction with PIVAS training	0.022	0.418	0.003	0.053	.958
Satisfaction with working in PIVAS	−2.782	0.364	−0.401	−7.652	.000
Need for scientific research	0.338	0.355	0.055	0.953	.341
Interest in scientific research	−0.529	0.369	−0.082	−1.432	.153
The ability to do scientific research	−0.311	0.319	−0.042	−0.977	.329

PIVAS = pharmacy intravenous admixture services.

## 4. Discussion

This multi-center cross-sectional survey investigated the status of depression and anxiety among PIVAS leaders and staff, and analyzed factors influencing anxiety and depression.

At present, more than 2000 medical institutions in China have established PIVAS,^[11]^ so 6.85% PIVAS in China participated in our study. The results showed that anxiety and depression were relatively common in PIVAS leaders and staff in China. The prevalence of anxiety among staff in this study was similar to that reported by Chao et al.^[7]^ Multiple linear regression showed that the degree of recognition by doctors, PIVAS charge standard, and PIVAS profit and loss situation were associated with mental health among PIVAS leaders. Satisfaction with working in PIVAS, job title, and working hours per day were associated with depression scores among PIVAS staff.

In terms of influencing factors, PIVAS leaders in situations with no charge standard, a low degree recognition by doctors, and without profit were likely to report symptoms of anxiety and depression. There are 2 possible reasons for these findings. First, in China, most PIVAS have no charge standard, and are therefore in a state of loss and need to be financially subsidized by the hospital^[3,12]^; this means these services operate under high pressure. Second, the service objects of PIVAS include patients, clinicians, and nurses. PIVAS must communicate with doctors frequently, especially when encountering unreasonable orders for interventions. This requires PIVAS staff to have solid professional knowledge and good communication skills, and means PIVAS leaders need to pay close attention to the development of ability among staff, which may create additional pressure for PIVAS leaders.^[13]^ Therefore, leaders are concerned about doctors’ recognition of PIVAS, which was therefore a factor influencing their mental health.

Staff that were not satisfied with working in PIVAS, had lower job titles, and who worked longer hours per day were more likely to report symptoms of anxiety and depression than other staff. This may be because PIVAS is responsible for the centralized configuration of intravenous medication for the whole hospital, and the configuration time is concentrated, meaning the workload is heavy and high-paced.^[6,8,13]^ Therefore, the longer hour staff work in PIVAS, the more likely they are to feel tired and less satisfied with work, which can affect their mental health. In addition, all staff in hospitals needs to publish research papers to promote their professional title,^[4]^ but the high workload in PIVAS leaves little time to write scientific research papers. As the individual’s professional title affects their income, employees with low professional titles are more likely to report symptoms of anxiety and depression.

The anxiety and depression status of PIVAS staff and leaders merits more attention from hospitals and society, and hospitals should implement measures to improve the mental health of PIVAS employees, such as strengthening vocational training, improving the sense of job acquisition and income level, and enriching the lives of staff to help them adjust to the work pressure.^[14]^ In addition, PIVAS could introduce automation and artificial intelligence equipment, which could improve work efficiency and reduce errors and work intensity, which may in turn reduce the incidence of depression and anxiety.^[6]^

Our study had several limitations that should be addressed in further studies. First, as we did not have a full list of PIVAS leaders and staff, we could not use random sampling to select participants. However, the sample size of this study was relatively large and the survey respondents were from different regions of China, which gave good representation. Second, the cross-sectional study design means we could not make causal inferences. Third, although GAD-7 and PHQ-9 have been used to detect symptoms of anxiety and depression disorders in various settings and across diverse populations in China,^[15–18]^ and which were with good reliability and validity, but anxiety and depression were measured by self-assessment, which might have resulted in an exaggerated incidence of mental health problems. Fourth, we only use the WeChat group to recruit participants, it would be better to utilize multiple methods to enhance generalizability. Further larger-scale studies with long follow-up periods are needed to continuously investigate the mental health status of PIVAS leaders and staff more comprehensively.

In conclusion, depression and anxiety are common among PIVAS leaders and staff in hospitals in China, and hospitals should implement measures to improve the mental health of PIVAS leaders and staff.

## Acknowledgements

We also thank Audrey Holmes, MA, from Liwen Bianji (Edanz) (www.liwenbianji.cn) for editing the language of a draft of this manuscript.

## Author contributions

**Conceptualization:** Lingli Zhang.

**Data curation:** Chunsong Yang, Yaya Yang, Lingli Zhang, Yong Hu.

**Formal analysis:** Chunsong Yang, Yaya Yang, Lingli Zhang, Yong Hu.

**Investigation:** Chunsong Yang, Dan Li.

**Methodology:** Chunsong Yang, Lingli Zhang, Dan Li.

**Resources:** Yaya Yang.

**Writing – original draft:** Yaya Yang, Lingli Zhang, Yong Hu.

**Writing – review & editing:** Yaya Yang, Lingli Zhang.
